# QRISK3 score is predictive of thrombotic risk in patients with myeloproliferative neoplasms

**DOI:** 10.1038/s41375-025-02681-9

**Published:** 2025-07-24

**Authors:** Andrea Duminuco, Raj Vaghela, Sukhraj Virdee, Claire Woodley, Susan Asirvatham, Natalia Curto-Garcia, Priya Sriskandarajah, Jennifer O’Sullivan, Hugues de Lavallade, Deepti Radia, Shahram Kordasti, Giuseppe A. Palumbo, Claire Harrison, Patrick Harrington

**Affiliations:** 1 Department of Haematology, Guy’s and St Thomas’ NHS Foundation Trust, London, UK; 2 Department of Medical, Surgical Sciences and Advanced Technologies “G.F. Ingrassia”, University of Catania, Catania, Italy; 3 School of Cancer and Pharmaceutical Science, King’s College London, London, UK

**Keywords:** Myeloproliferative disease, Myeloproliferative disease, Risk factors

## Abstract

Thromboembolic events (TE) represent the commonest cause of morbidity and mortality in polycythemia vera (PV) and essential thrombocythaemia (ET). The QRISK3 model is a tool for predicting TE in the general population, with 7.5% recognised as a threshold to identify high-risk patients. We analyzed data of 937 patients (490 ET and 447 PV) with a median follow-up of 85 and 95 months, reporting an occurrence of 52 and 73 TE, respectively. Median QRISK3 scores at diagnosis were higher in conventional high-risk patients in both cohorts (ET; 4.2 in high-risk vs. 2.4 in low-risk, PV; 8.8 vs. 2.8, p < 0.001). During follow-up, a QRISK3 score greater than 7.5%, demonstrated potential to further stratify individuals at high risk of TE, outperforming standard risk assessments in both low and high-risk patients. Using cytoreductive treatment instead of active surveillance in patients with QRISK3 ≥ 7.5% conferred a reduced risk of thrombosis in both cohorts. Of this group, 79.7% with ET and 86.9% with PV, on cytoreductive therapy, remained thrombosis free, compared with 64.1% and 57.1% of those not receiving cytoreductive therapy (p = 0.018/0.034). QRISK3 identifies patients in whom cytoreductive therapies may be indicated, and provides a tool that allows patients to assess, monitor and reduce their cardiovascular risk.

## Introduction

Myeloproliferative neoplasms (MPN) are haematopoietic stem cell disorders characterized by uncontrolled cellular growth, with overproduction of differentiated blood cells, associated with specific somatic mutations in the JAK-STAT pathway genes [1]. The most common entities are essential thrombocythemia (ET), polycythemia vera (PV), and myelofibrosis (MF).

In patients with MPN, the occurrence of thrombotic events (TE) remains the primary cause of both morbidity and mortality [2]. As widely reported, several mechanisms are involved in the increased thrombotic risk. The hypercoagulable state derives from a complex interplay of factors including endothelial alterations, higher platelet count and increased blood viscosity, all of which promote thrombus formation.

Traditionally ET and PV patients are primarily stratified according to future risk of TE using age (lower or higher than 60/65 years) and history of previous thrombotic events [3, 4] In ET, the revised IPSET-thrombosis model is an additional tool that predicts probability of thrombotic events and considers the presence of the *JAK2*V617F mutation versus other driver mutations, age, and previous thrombotic events [5, 6]. The precise role of concurrent cardiovascular comorbidities, such as diabetes mellitus, hypertension, obesity, hypercholesterolemia and smoking habit, which are known to increase cardiovascular risk, have not been fully assessed. As such, to date, they have not been included in most thrombotic prognostic models used for ET or PV [7].

The QRISK3 score has emerged as a powerful predictor of cardiovascular risk in the general population [8]. It is validated in a cohort of almost 3 million UK subjects, with an algorithm designed to estimate an individual’s risk of experiencing a cardiovascular event over a period of ten years in people aged 25–84, without a prior cardiovascular event. It incorporates various clinical and demographic variables, such as age, history of hypertension, atrial fibrillation, and severe mental illness, and high BMI, comprehensively evaluating an individual’s risk profile. It was recently studied in a cohort of 438 patients with MPN but requires further validation [9].

Recently, How and colleagues reported that the presence of at least one cardiovascular risk factor in MPN patients increases the risk of thrombosis (HR 3.05; 95% CI 2.39–3.92) and reduces the overall survival (HR 2.52; 95% CI 1.9–3.35) in MPN [10], but clinical studies that assess the role of cardiovascular comorbidities in thrombotic risk in this population are lacking. In view of this, we evaluated the potential role of QRISK3 score for determining thrombotic risk in ET and PV patients at diagnosis and dynamically during follow-up.

## Material and methods

### Aim and study design

We retrospectively reviewed the use of the QRISK3 score to assess cardiovascular risk in a large single tertiary centre patient population, aiming to identify patients conventionally stratified as low-risk for TE, who may benefit from cytoreductive intervention.

This retrospective study included 937 patients affected by ET or PV, managed in the Department of Haematology at Guy’s and St Thomas’ (GSTT) NHS Foundation Trust, London, UK. Patients were required to have at least 3 months of follow-up, and available data for calculating the QRISK3 score. The diagnosis of MPN was made according to International Consensus Criteria/WHO 5^th^ edition criteria [11, 12]. Patients were stratified as low- and high-risk according to age and previous TE. For ET, the IPSET-revised score was also evaluated.

Data were manually collected using the Electronic Patient Record (EPR – iSOFT Clinical Manager), the electronic health record (EHR) used at GSTT, where patient information, clinical documentation, test results, referrals, electronic discharge letters, and clinic outcome letters are reported. The QRISK3 score was evaluated at diagnosis and at the time of TE where possible, using a web tool (https://qrisk.org/index.php, accessed on April 10, 2024). For a single patient older than 84, we assumed this age for QRISK3 score calculation. Patients were stratified firstly according to standard classification (age ≥65 years old and history of TE), then considering QRISK3 score, with the aim of identifying a threshold at diagnosis and during follow-up that might predict further TE risk. As per international cardiovascular guidelines, including the 2021 European Society of Cardiology guidelines, a QRISK3 score of ≥7.5% risk for TE was used to stratify low and high-risk patients [13–15]. A TE was defined as any event that occurred regardless of severity, with grade 1 (usually asymptomatic and an incidental finding) to grade 5, according to CTCAE.

### Statistical analysis

Baseline clinical characteristics were summarized in counts and percentages. Shapiro-Wilks test was used to verify normality across the distribution. The difference between the groups was assessed using the Mann–Whitney U test or Kruskal–Wallis test for continuous data and the chi-square test or Fisher exact test for categorical data. Receiver operating characteristic (ROC) analysis was performed to assess the role of QRISK3 in predicting TE at diagnosis, while the best threshold was identified through optimal cut-point value.

A p-value less than 0.05 was considered significant. MedCalc Statistical Software version 19.2.6 (MedCalc Software bv, Ostend, Belgium; https://www.medcalc.org; 2020) and Prism 9 (version 9.5.1, 528, January 24, 2023) were used for statistical analysis.

### Ethical approval

The study was performed in accordance with U.K. research regulations and was approved by an external ethical committee (reference 23/NW/0105).

## Results

Clinical data from 490 ET and 447 PV patients were assessed. Median ages were 47 [25–85] and 46 years [25–84], respectively. Disease-specific characteristics, the last therapeutic approach at censoring (or last follow-up), and the outcome (censored or not, myelofibrotic/acute myeloid leukaemia transformation) are summarized (Table 1). For ET, 389 patients were still alive, while 76 were lost to follow-up, with a median follow-up of 85 months across all ET patients. For PV, 382 were alive, and 49 lost to follow-up, with overall median follow-up of 95 months. 18 patients progressed to post-ET (PET) MF (9) and post-PV (PPV) MF (9). According to standard guidelines, at diagnosis, 355 (72.4%) ET patients, and 312 (69.8%) with PV were classified as low-risk. Additionally, for 424 ET patients with driver mutation data available, the IPSET-revised score identified 138 (32.6%) very-low, 174 (41%) low, 21 (4.9%) intermediate, and 91 (21.5%) high-risk patients (Supplementary Results 1). The median baseline QRISK3 score was similar in both conditions; 3.2% for ET and 3.5% for PV (p = 0.18).

**Table 1:** Clinical data of 937 PV and ET patients.

	ET patients (490) [range] – (%)	PV patients (447) [range] – (%)
Sex, male/female	145/345 (26/74)	214/233 (48/52)
Age at diagnosis, years	47 [25–85]	46 [25–84]
Mutational status:	Available for 424	Available for 420
JAK2	253 (59.7)	405 (96.4)
MPL	7 (1.6)	/
CALR	117 (27.6)	/
Triple-negative	47 (11.1)	15 (3.6)
VAF, %	*Available for 34* 13 [1–41] % ^a^	*Available for 39* 39 [7–98] %**
Risk at diagnosis		
Low	355 (72.4)	312 (69.8)
High	135 (27.5)	135 (31.2)
QRISK3 score at diagnosis	3.2 [0.1–40.6] %	3.5 [0.1–41] %
Primary MPN-specific approach*		
Watch and wait/venesection	292 (59.5)	276 (61.7)
Hydroxyurea	143 (29.2)	106 (23.7)
Ruxolitinib	11 (2.3)	10 (2.2)
Interferon-α	44 (9)	55 (12.4)
Thrombosis history at/before diagnosis	64 (13.1)	84 (18.8)
Thrombosis during follow-up		
Events	52 events in 46 patients (9.4)	73 events in 62 patients (13.9)
Time to thrombosis, months	39 [2–324]	38 [1–232]
Thrombosis site:		
Pulmonary embolism	2 (3.8)	5 (6.8)
Myocardial infarction	8 (15.4)	16 (21.9)
Cerebrovascular accident	14 (26.9)	27 (37.1)
Portal vein	4 (7.7)	5 (6.8)
Other VTE	23 (44.3)	20 (27.4)
Other arterial	1 (1.9)	/
Transformed to PET/PPV MF	9 (1.8)	9 (2)
Status:		
Alive	389 (79.4)	382 (85.4)
Death	25 (5.1)	16 (3.6)
Lost at follow-up	76 (15.5)	49 (11)
Median follow-up, months	85 [3–481]	95 [3–405]

PET post ET, PPV post PV; MF myelofibrosis.

*therapy used from diagnosis or for longest time period if different.

*NA* not available, *VTE* venous thromboembolism.

### Thrombosis occurrence

#### ET cohort

During follow-up, 46 patients experienced TE, with 52 events overall, after a median of 39 months from diagnosis [2–324].

**Fig. 1:**
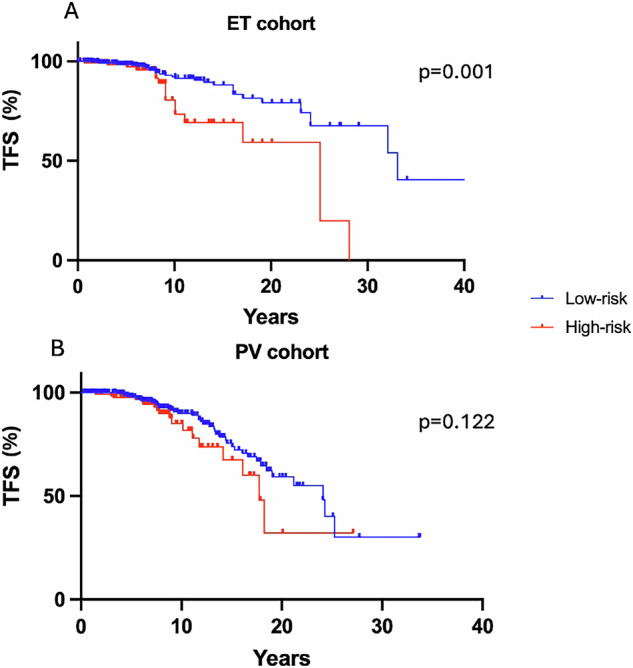
Thrombosis rate in conventional thrombotic risk groups. **A**, **B** Incidence of thrombosis in ET and PV patients, occurring in conventional high-risk (HR) and low-risk (LR) groups.

They were divided into 23 (44%) arterial events (cerebrovascular accidents n = 14, myocardial infarctions n = 8, and retinal artery occlusion n = 1) and 29 (56%) venous (n = 4 portal vein, n = 2 cerebral venous and pulmonary embolism, n = 21 other venous thromboembolism). 17 patients were classified as high-risk due to age and history of previous TE, with 15 of these on cytoreductive treatment including hydroxycarbamide (n = 12), ruxolitinib (n = 2), or interferon-α (n = 1). 5 were also receiving anticoagulant therapy (DOAC or VKA) at the time of event. Conversely, 29 low-risk patients, with a median age of 44 [25–64] years, experienced TE with a median time from diagnosis of 44 [4–113] months; at TE, 14 were on cytoreductive treatment, while 15 on active surveillance. There was a significant difference in the cumulative incidence of thrombotic events between conventionally high and low-risk groups over time (17/135 vs 29/355, p = 0.001, Fig. 1A). The median QRISK3 score for low-risk patients that experienced TE was 12.1% [0.9–43.9].

#### PV cohort

For the PV cohort, there were 73 TE (higher than ET cohort, p = 0.032), occurring in 62 patients, with a median time from diagnosis of 38 months [1–232]. 48 (65.8%) were arterial events (cerebrovascular accident n = 27, myocardial infarction n = 16, pulmonary embolisms n = 5) and 25 (34.2%) were venous (splenic/portal vein n = 5, cerebral venous n = 3, other venous thromboembolism n = 17).

Of PV patients with TE, 18 were classified as high-risk due to age or previous TE. The remaining 44 low-risk patients experienced TE (median age of 42 [25–64] years), with a median time from diagnosis to the event of 58 months and had a median QRISK3 score of 6.5% [1.7–26.7]. For this cohort, there was no significant difference observed in the occurrence of thrombosis between conventional high and low-risk groups (p = 0.122, Fig. 1B). Among the low-risk PV group, 19 were managed with active surveillance/venesection, and others on cytoreductive treatment (hydroxycarbamide, ruxolitinib, or interferon-α). Anticoagulant therapy with DOAC or VKA associated with aspirin was administered only in patients with prior thrombotic events.

## QRISK3 score

**Fig. 2:**
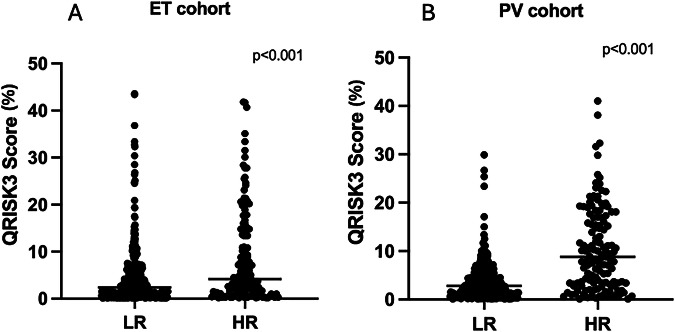
QRISK3 score in conventional thrombotic risk groups. QRISK3 score evaluated at diagnosis, divided for low (younger than 65 years old) and high-risk (older than 65 and patients with a history of TE at/prior diagnosis) in ET (**A**) and PV (**B**) cohorts. LR low-risk, HR high-risk.

QRISK3 score, aligned with conventional risk stratification, and was significantly higher in patients older than 65 and/or with a history of thrombosis in the ET (4.2% vs. 2.4%, p < 0.001, Fig. 2A) and in the PV cohorts (8.8% vs. 2.8%, p < 0.001, Fig. 2B).

**Fig. 3:**
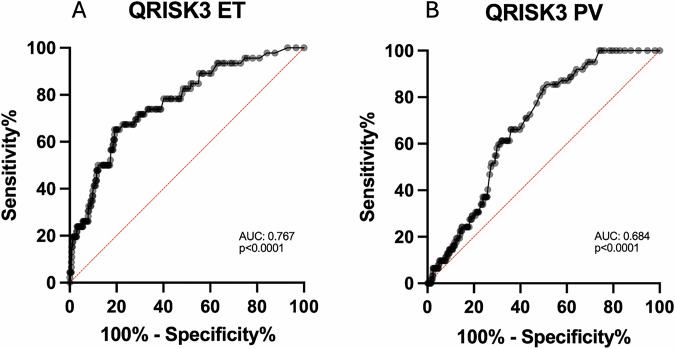
Analysis of the accuracy of a QRISK3 score of ≽7.5% to predict thrombotic risk. **A**, **B** ROC curves evaluating a threshold for QRISK3 of 7.5% for the ET cohort with standard error 0.04, confidence interval (CI) 95%, 0.694–0.839 (**A**), and for PV cohort with standard error 0.02, CI 95%, 0.717–0.809.

Based on ROC analysis (Fig. 3A, B), QRISK3 of 7.5%, resulted in a sensitivity of 65% (confidence interval [CI] 95%, 51–77%) and a specificity of 81% (CI 95%, 77–84%) in predicting the occurrence of TE across the whole ET cohort. For PV patients, a threshold of 7.5% showed a sensitivity of 42% (CI 95%, 30–54) and specificity of 78% (CI 95%, 73–82). The QRISK3 threshold of 7.5% was applied in the analysis described below. The optimal threshold in PV for improving accuracy was >5.5% (sensitivity 79%, specificity 70%). Overall, 118 (24.1%) ET patients and 112 (25.1%) PV patients had a QRISK3 score ≥7.5%.

**Fig. 4:**
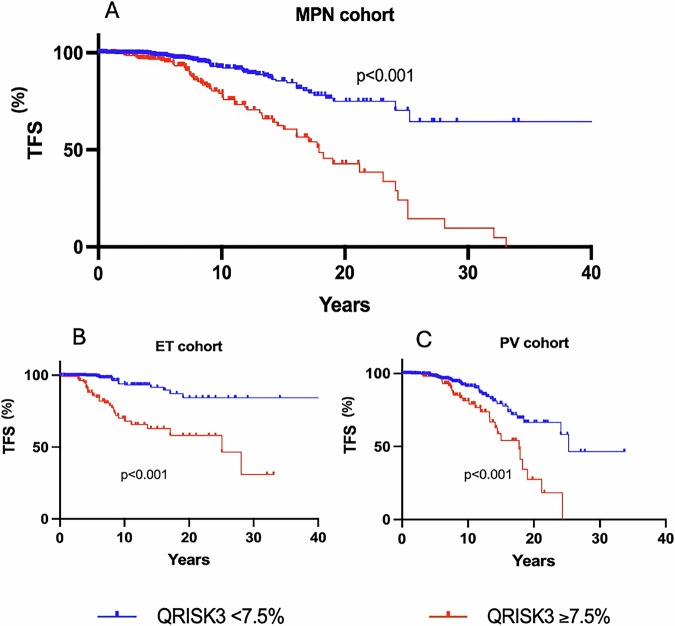
Analysis of thrombosis rate using QRISK3 score. Stratification according to QRISK3 of ≽ or <7.5% for (**A**) the whole MPN cohort (**B**) the essential thrombocythaemia cohort and (**C**) the polycythaemia vera cohort.

First, taking the total MPN cohort, with ET and PV patients combined, a QRISK3 score >7.5% showed significantly reduced TFS, confirming its potential importance (Fig. 4A). This threshold was also able to identify patients with significantly reduced TFS when assessing ET and PV cohorts separately (Fig. 4B, C).

**Fig. 5:**
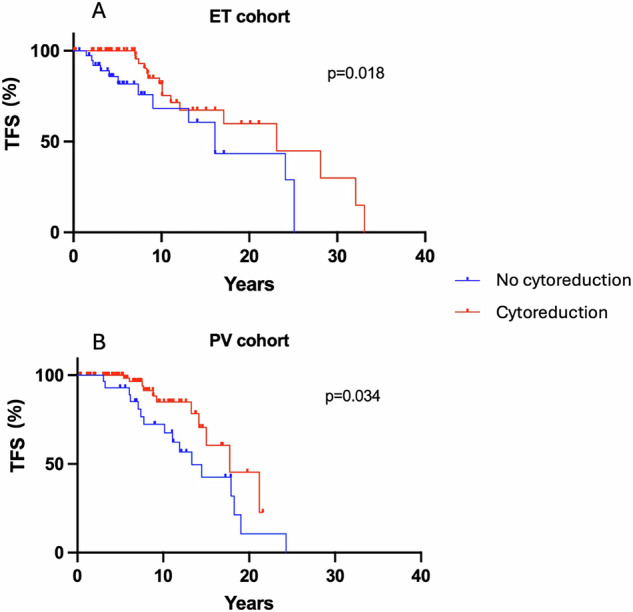
Impact of cytoreductive therapy on thrombosis in those with QRISK3 score ≽7.5%. **A**, **B** Differences in risk of thrombosis for essential thrombocythemia (ET) and polycythemia vera (PV) patients with QRISK3 ≥ 7.5 between those receiving cytoreductive treatment and those who were not.

We then evaluated if active cytoreductive treatment is associated with reduced incidence of thrombosis, in patients classified as high risk according to QRISK3 score. Overall, at last follow-up, 291 PV and 248 ET patients were receiving cytoreductive treatment, as detailed in Table 1. Across the whole cohort of ET patients with QRISK3 ≥ 7.5%, 30 (25.4%) patients experienced a TE during follow-up. The presence of individual conditions recognised to increase cardiovascular risk, such as diabetes mellitus, hypertension, smoking status (all with p > 0.99), hypercholesterolemia (p = 0.68), obesity (p = 0.729), or gender (p = 0.828), did not demonstrate an increased risk of thrombosis in this analysis. Older age, as expected, conferred a higher risk of TE, with a median age of patients who had TE of 58 compared with 45 years for those who did not, p = 0.001. As expected, cytoreductive treatment reduced the risk of thrombosis. This was administered in 79 patients with QRISK ≥ 7.5%, with 63 (79.7%) remaining thrombosis-free, superior thrombosis-free survival to those patients not on cytoreductive therapy who had QRISK ≥ 7.5% (TE occurred in 25 out of 39, p = 0.018, Fig. 5A).

Finally, with regards to the IPSET-revised score, 26 out of 312 (8.3%) very low/low-risk patients experienced a TE, which in our cohort was not significantly different from those with intermediate/high risk (9 out of 112, 8%, p = 0.922). QRISK3 improved conventional risk assessment using the IPSET-revised score, particularly in patients with very low/low risk where, overall, 19 out of 54 (35.2%) patients with QRISK3 ≥ 7.5% had a TE, compared to 7 out of 251 with QRISK3 < 7.5% (p < 0.001, Supplementary Results 1).

In the PV cohort, 27 (24.1%) patients with QRISK3 ≥ 7.5% reported a TE after diagnosis. No statistical differences in terms of risk were described for sex (p = 0.508), diabetes mellitus (p = 0.757), hypercholesterolemia (p = 0.456), smoking status (p = 0.53), obesity (p = 0.209), and hypertension (p = 0.08), while, as seen with ET, age increased thrombotic risk (67 for those who experienced a TE vs. 60 years for thrombosis-free, p < 0.001). Active cytoreductive treatment again demonstrated a protective effect against thrombosis. 73 of 84 (86.9%) patients with QRISK3 ≥ 7.5% that received cytoreductive therapy remained thrombosis-free, while 16 of 28 (57.1%) with QRISK3 ≥ 7.5% who were not receiving cytoreductive treatment experienced a TE during follow-up (p = 0.034, Fig. 5B).

**Fig. 6:**
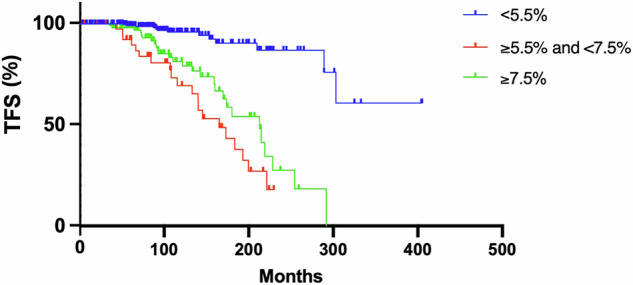
Analysis of thrombosis rate in PV using QRISK3 score. Thrombosis free survival (TFS) in patients with polycythaemia vera (PV) categorised based on QRISK3 score of either <5.5%, >5.5% and <7.5%, or ≥7.5%.

Finally, in view of the ROC analysis identifying a lower optimal threshold ( > 5.5%) in the PV cohort, we then assessed QRISK3 score focusing on this threshold as well as those with QRISK3 between the two thresholds. 54 patients reported a QRISK3 ≥ 5.5% and <7.5%. All were <65 years old, and 8 (14.8%) were classified as conventionally high-risk for a history of TE. In this subgroup, thrombosis was overall reported in 3 out of 8 (37.5%) conventional high-risk and 19 out of 46 (41.3%) low-risk patients. Median thrombosis-free-survival was not reached for those with QRISK3 < 5.5%, 165 months for those between 5.5% and 7.5%, and 213 months in those ≥7.5%, suggesting again that the lower threshold of <5.5% is more able to accurately stratify the PV cohort (Fig. 6).

**Fig. 7:**
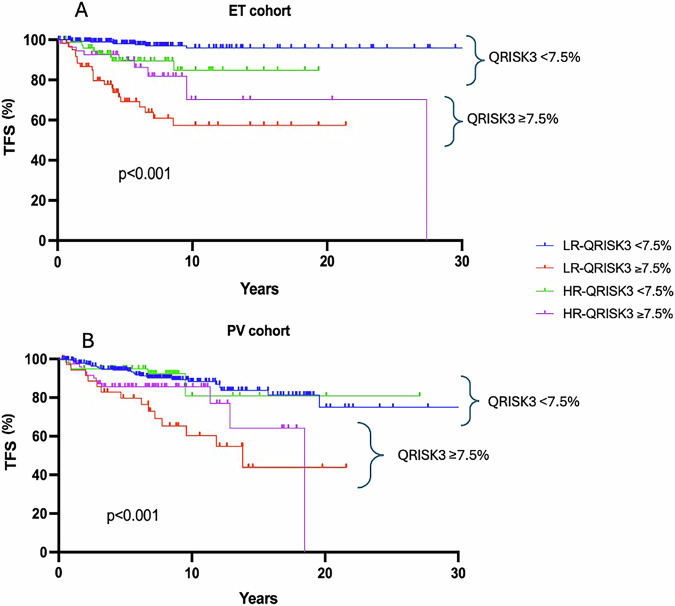
Analysis of thrombosis rate combing both QRISK3 score and conventional assessment. **A**, **B** Patients stratified according to both conventional factors (age and previous thrombotic event) and QRISK3 > 7.5% in essential thrombocythemia (ET) and polycythemia vera (PV), demonstrating the potential role for QRISK3 to identify patients with high thrombotic risk. TFS thrombosis free survival, LR low risk, HR high risk.

We then stratified patients according to both conventional risk factors and QRISK3. In ET, 8 of 293 patients who were low-risk for both conventional factors and QRISK3 score experienced a TE. 21 of 62 who were conventionally low-risk but had QRISK ≥ 7.5%, 8 of 79 who were conventionally high-risk but had QRISK3 < 7.5%, and 9 of 56 who were high-risk for both, experienced a TE. This approach demonstrated that QRISK3 score significantly improves the stratification of patients (p < 0.001, Fig. 7A and Supplementary File 1).

In PV, 30 of 276 conventionally low-risk patients also with QRISK3 score <7.5% experienced a TE. 14 of 36 conventionally low-risk but with QRISK3 ≥ 7.5%, 5 of 59 conventionally high-risk with QRISK3 < 7.5%, and 13 of 76 who were high-risk according to both models experienced a TE. This again demonstrated that QRISK3 outperforms standard stratification methods (p < 0.001, Fig. 7B and Supplementary File 2).

## Subanalysis in conventional low risk patients

In the ET cohort, 412 (84.1%) patients were younger than 65 years old at diagnosis (median age 44 years, ranging from 25–64). This included 355 (86.2%) cases classified as low-risk and 57 (13.8%) with prior/concurrent thrombosis, thus considered as high-risk. The median QRISK3 score from the low-risk cohort was 2.7% (range 0.1–43.6), compared with 7.1% (range 0.2–41.8) in those aged <65 years with a history of TE (p < 0.001, Supplementary Fig. 1A). Excluding patients with a history of thrombosis, and thus considering only conventional low-risk patients, 17.5% (62) of patients, reported a score ≥7.5%, and, could be categorised as high-risk using QRISK3.

Overall, regarding patients classified as low-risk at diagnosis who went on to have a TE during follow-up, a QRISK3 threshold of 7.5% identified those at increased risk. Of 355 patients in this subgroup, 30 of 62 with baseline QRISK3 ≥ 7.5% compared with 16 of 293 with QRISK3 < 7.5% had a TE (p < 0.001). There was no significant difference in time to thrombosis, with these occurring at a median time from diagnosis of 31 [2–324] in QRISK3 < 7.5% vs. 41 [8–113] months in ≥7.5% (p = 0.824). At TE, 5 out of 30 and 2 out of 16 were ≥65 years old, thus becoming high-risk. The median QRISK3 score at the time of TE in this sub-cohort was 8.5% [3.5–12.5, with 11 of 16 patients ≥7.5%]. No differences were reported concerning arterial or venous events (p > 0.05).

Regarding the PV cohort, 388 (86.7%) patients were aged <65 years at diagnosis (median age of 44, 25–64). 312 (80.4%) were stratified as low-risk, while the remaining patients experienced a TE. The median QRISK3 score was 2.8% [0.1–29.9] for patients without TE, and 3.4% [0.1–19] for those who experienced thrombosis (p = 0.044, Supplementary Fig. 1B).

Excluding patients with a history of TE, 11.5% (36) of 312 patients younger than 65 had a QRISK3 score ≥7.5% at diagnosis. In this subset of conventionally defined low-risk patients, QRISK3 ≥ 7.5% calculated at diagnosis was able to identify patients with an increased risk of TE during follow-up (14 of 36 with QRISK3 ≥ 7.5% compared with 30 of 276 < 7.5%, p < 0.001). The median QRISK3 score at the time of thrombosis was 8.3% [2.3–26.7], with 24 out of 44 patients scoring ≥7.5%.

Finally, to confirm that QRISK3 assessment also remains valid using thrombotic risk stratification based on an age threshold of ≥60 years old, 325 (66.3%) patients in ET and 290 (64.9%) in PV belonged to the low-risk group of <60 years of age and absence of prior thrombosis. Of these groups, 18 of 50 (36%) ET patients and 8 of 25 (32%) PV patients with QRISK3 ≥ 7.5%, experienced a TE during follow-up, higher than those with QRISK3 < 7.5% (8 out of 275, p < 0.001, 30 out of 265, p = 0.038 respectively), suggesting again that the early initiation oof cytoreductive treatment should be considered in this patient group (Supplementary Fig. 2).

## Discussion

Despite advancements in stratifying thrombotic risk in the management of MPN patients, existing models do not account for the impact of cardiovascular comorbidities, which play a critical and modifiable role in shaping individual patient risk.

In the pivotal ECLAP study, cardiovascular mortality accounted for 45% of all deaths in PV patients, with an incidence rate of 1.7/100 person/year demonstrating the increased risk faced [3]. Recent reports have shown an incidence rate of thrombosis following diagnosis of 2.62/100 person-years, with results comparable to the findings of CYTO-PV clinical trial (2.7/100 patient-years) [13]. In a Mayo cohort, with median follow-up of 109 months in PV, 128 patients out of 587 (22%) experienced thrombotic complications. History of prior events, hyperlipidaemia, and hypertension (all with p ≤ 0.03) were significant predictors for subsequent arterial events on multivariate analysis. Prior events, a leucocyte count ≥11 × 10^9^/L, and experiencing major bleeding were identified as predictors for subsequent venous events (all with p ≤ 0.05) [14]. Moreover, in a more extensive study of Italian haematology centres belonging to GIMEMA (Gruppo Italiano Malattie Ematologiche dell’Adulto), 235 patients with PV and 259 with ET were retrospectively evaluated, with an occurrence of thrombosis observed in 20.4% and 13%, respectively. Between the CV risk factors, smoking was reported in 14.3%, hypertension in 46.5%, hypercholesterolemia in 12.5%, and DM in 8.2%, with results similar to that described in our study [15]. Lastly we have previously highlighted the impact of hypertension as a specific risk factor for thrombotic events in MPN patients in a smaller study utilizing a natural language processing approach [16].

These findings underline the necessity for novel predictive tools that integrate traditional risk factors alongside a broader spectrum of cardiovascular comorbidities. Such tools should provide a more comprehensive and dynamic assessment, enabling personalized management strategies to mitigate thrombotic risk and improve patient outcomes.

We demonstrate here as a novel finding, that the QRISK3 model can be used in PV or ET patients classified as high and low-risk, using conventional scores, to identify those who have an increased risk of experiencing a TE [17]. Improved stratification of conventional low-risk patients is potentially the area in which QRISK3 assessment is most beneficial, in order to determine which additional patients would benefit from cytoreductive therapy. This is supported by our sub-analysis in ET patients showing significantly increased rates of thrombosis in those with QRISK3 > 7.5% in the IPSET-revised low and very low risk groups. Both at diagnosis and during follow-up, at the time of thrombosis, the QRISK3 threshold of >7.5% was able to improve thrombotic risk stratification, when compared with factors conventionally used. Whilst the ROC analyses confirmed the validity of the 7.5% threshold in ET, the optimal threshold value in PV was lower at 5.5%, in keeping with a higher incidence of thrombotic events in PV. QRISK3 score can best be considered as an adjunct to conventional assessment and patients with prior thrombosis should continue to be considered high-risk for future events, regardless of QRISK3 score. Significantly, our data also indicates that cytoreductive therapy reduces TE incidence in patients stratified as high-risk according to QRISK3 score. This supports our assumption that these patients should be considered for active intervention to reduce their associated thrombotic risk, in addition to addressing modifiable factors identified using QRISK3 analysis.

Limitations to our work include the retrospective nature of the study, with the analyses performed considering the primary treatment or therapy at time of thrombosis, and not considering the sequence of different treatments administered during follow-up. Additionally, for some patients the TE occurred after an extended follow-up when the QRISK3, being a dynamic score primarily influenced by age, was significantly higher (median 8%). We recognise the patient cohort’s single-centre nature, and our cohort’s median age is notably younger than would be expected for ET and PV, which may reflect the status as a tertiary referral centre. We also recognise that the age threshold of >65 years to define high-risk disease, which was primarily used, whilst being recognised in the country where the study was performed, does not align with the lower cut-off of >60 years that is commonly used in other countries [18]. Subanalysis of low-risk ET and PV patients also confirm the applicability of QRISK3 assessment when a 60-year-old threshold is used to determine high risk patients. In the future, roles for other variables, such as accessory mutations and the allelic ratio of driver mutations, may be demonstrated to play a role in causing TE [17, 19].

These findings support the use of QRISK3 as a tool that serves not only as a predictor of thrombotic risk at diagnosis, but also as a valuable instrument for longitudinal risk assessment. Further studies are ongoing, aiming to clarify its role prospectively during follow-up, with regular monitoring of the QRISK3, which could enable clinicians to identify dynamic changes in a patient’s risk profile. This would potentially allow for early and tailored interventions in those transitioning into higher-risk categories and improve patient outcomes by addressing emerging risks before TE occur. Finally, the use of an easily self-assessed web tool could allow patients to independently calculate their own QRISK3 score, which may help motivate them to adopt lifestyle changes to reduce their cardiovascular risk.

In summary, cardiovascular comorbidities should be considered when assessing thrombotic risk in MPN patients, and clinicians should strive to modify these conditions where possible. QRISK3 score appears to be a valid tool that can be applied successfully in PV and ET to help improve thrombotic risk assessment, suggesting the utility of active cytoreductive treatment in high-risk patients.

## Supplementary information

Supplemental Material

## Data Availability

Data that support the findings of this study are available from the corresponding author, [PH], upon reasonable request.
