# Supplementary material for: QRISK3 score is predictive of thrombotic risk in patients with myeloproliferative neoplasms
Source: Leukemia. 2025 Jul 24;39(10):2384–90. doi: 10.1038/s41375-025-02681-9 (PMC12463673; doi:10.1038/s41375-025-02681-9)
Supplement: Supplementary file 1 — Supplemental Material [file 41375_2025_2681_MOESM1_ESM.docx]

**SUPPLEMENTARY FILES**

***Supplementary File 1.*** *Comparison between IPSET and QRISK3, evidencing how a QRISK3 ≥7.5% can address better higher risk patients in essential thrombocythemia.*

|  | Thrombosis free  (%) | Thrombosis occurrence (%) | Median QRISK3 score | p | Pts with QRISK3 ≥7.5% | p |
| --- | --- | --- | --- | --- | --- | --- |
| Revised IPSET score  Very low (138)  Low (174)  Intermediate (21)  High (91) | 129 (93.5)  157 (91.1)  20 (95.2)  73 (91.2) | 9 (6.5)  17 (9.8)  1 (4.8)  8 (8.8) | 2.8 vs 24.7  2 vs 9.2  /  4.8 vs 10.1 | <0.001  <0.001  /  0.213 | 20 (12 vs 8)  34 (23 vs 11)  7 vs 0  35 vs 7 | <0.001  <0.001  /  0.058 |

***Supplementary File 2****. Comparison in the occurrence of thrombotic events in essential thrombocythemia between groups split according to standard model risk (prior thrombotic event and age ≥65 years old) and QRISK3 ≥7.5% through Dunn’s multiple comparison test*

| Dunn's multiple comparisons test | Mean rank diff, | Significant? | Summary | Adjusted P Value |
| --- | --- | --- | --- | --- |
| LR-QRISK <7.5% vs. LR_QRISK ≥7.5% | -76,29 | Yes | **** | <0,0001 |
| LR-QRISK <7.5% vs. HR-QRISK <7.5% | -18,12 | No | ns | 0,2741 |
| LR-QRISK <7.5% vs. HR-QRISK ≥7.5% | -32,69 | Yes | * | 0,0104 |
| LR_QRISK ≥7.5% vs. HR-QRISK <7.5% | 58,17 | Yes | **** | <0,0001 |
| LR_QRISK ≥7.5% vs. HR-QRISK ≥7.5% | 43,61 | Yes | ** | 0,0057 |
| HR-QRISK <7.5% vs. HR-QRISK ≥7.5% | -14,56 | No | ns | >0,9999 |

*Supplementary File 3. Comparison in the occurrence of thrombotic events in polycythaemia vera between groups split according to standard model risk (prior thrombotic event and age ≥65 years old) and QRISK3 ≥7.5% through Dunn’s multiple comparison test*

| Dunn's multiple comparisons test | Mean rank diff, | Significant? | Summary | Adjusted P Value |
| --- | --- | --- | --- | --- |
| LR-QRISK <7.5% vs. LR_QRISK ≥7.5% | -55,47 | Yes | *** | 0,0001 |
| LR-QRISK <7.5% vs. HR-QRISK <7.5% | 4,337 | No | ns | >0,9999 |
| LR-QRISK <7.5% vs. HR-QRISK ≥7.5% | -14,42 | No | ns | 0,9505 |
| LR-QRISK ≥7.5% vs. HR-QRISK <7.5% | 59,81 | Yes | ** | 0,0011 |
| LR-QRISK ≥7.5% vs. HR-QRISK ≥7.5% | 41,05 | Yes | * | 0,0398 |
| HR-QRISK <7.5% vs. HR-QRISK ≥7.5% | -18,76 | No | ns | >0,9999 |

**Supplementary Figure 1**


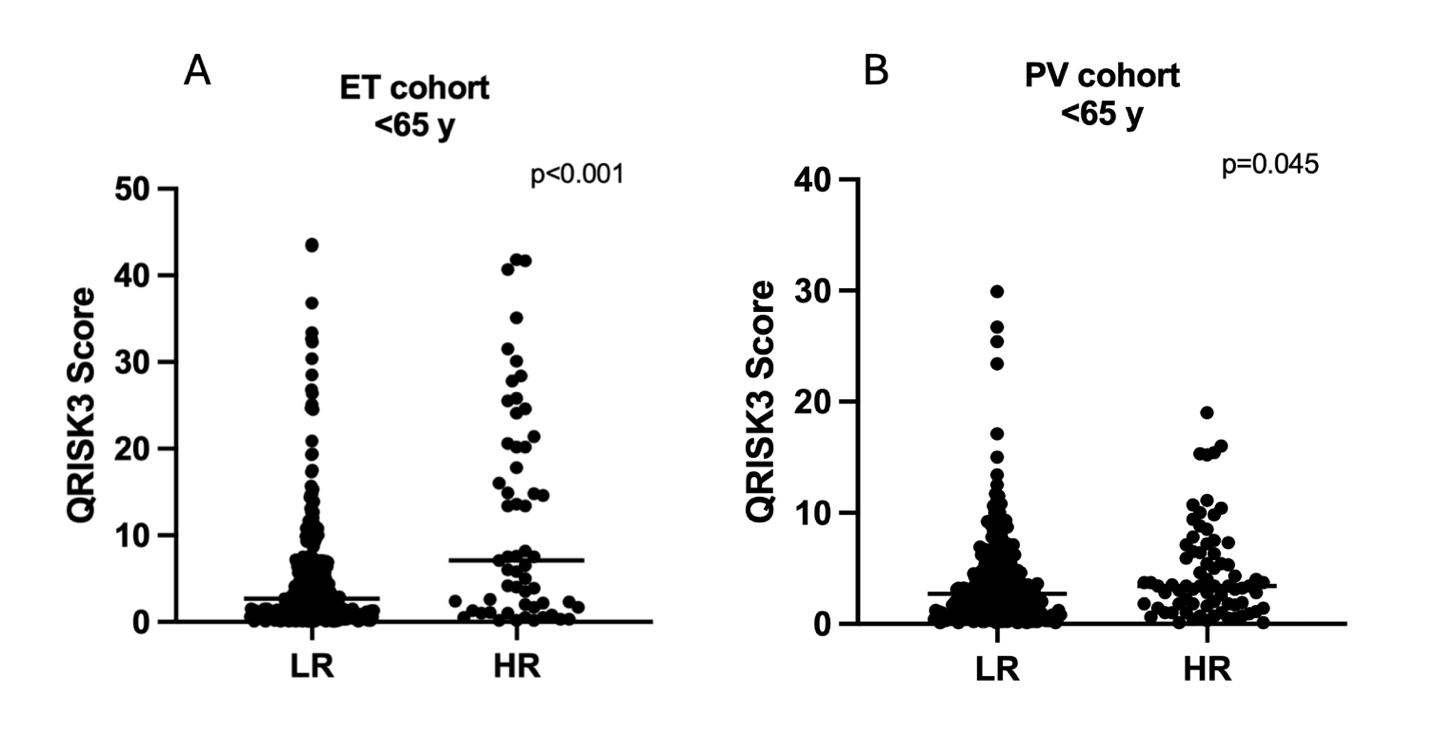


*Supplementary Figures 1A and B. QRISK3 score in patients <65 years categorised based on presence of conventional risk factors (HR [high-risk] – prior thrombotic events ) and (LR [low-risk] - absence of prior thrombotic events) ET (A, p<0.001) and PV (B, p=0.045) younger than 65 years old*

**Supplementary Figure 2**

*Supplementary Figures 2A and B. Stratification of ET (A) and PV (B) patients according to QRISK3, lower or higher than 7.5%, when high- and low-risk patients are considered based on the threshold of 60 years old*

**
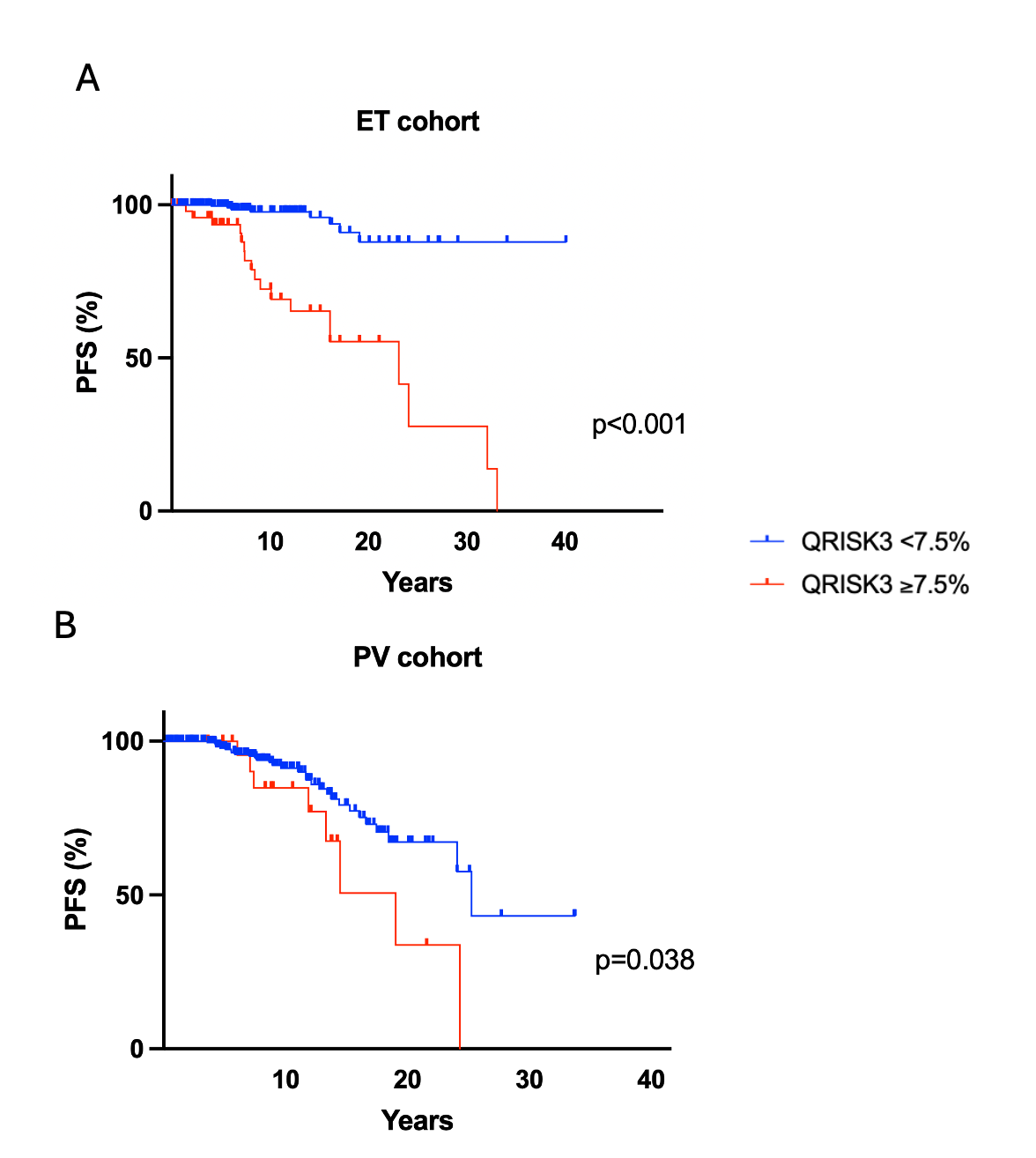
**
